# Development and External Validation of a Combined Clinical–Radiomic Model for Predicting Insufficient Hypertrophy of the Future Liver Remnant following Portal Vein Embolization

**DOI:** 10.1245/s10434-024-16592-z

**Published:** 2024-12-10

**Authors:** Qiang Wang, Torkel B. Brismar, Dennis Björk, Erik Baubeta, Gert Lindell, Bergthor Björnsson, Ernesto Sparrelid

**Affiliations:** 1 Department of Clinical Science, Intervention and Technology (CLINTEC), Division of Medical Imaging and Technology, Karolinska Institutet, Stockholm, Sweden; 2 Department of Radiology, Karolinska University Hospital Huddinge, Stockholm, Sweden; 3 Department of Surgery, Linköping University Hospital, Linköping, Sweden; 4 Department of Imaging and Functional Medicine, Skåne University Hospital, Lund, Sweden; 5 Diagnostic Radiology, Department of Translational Medicine, Lund University, Malmö, Sweden; 6 Department of Surgery, Skåne University Hospital Comprehensive Cancer Center, Clinical Sciences Lund, Faculty of Medicine, Lund University, Lund, Sweden; 7 Division of Surgery and Oncology, Department of Clinical Science, Intervention and Technology, Karolinska Institutet, Karolinska University Hospital, Stockholm, Sweden

**Keywords:** Portal vein embolization, Radiomics, Future liver remnant, Machine learning, Liver cancer

## Abstract

**Objectives:**

This study aimed to develop and externally validate a model for predicting insufficient future liver remnant (FLR) hypertrophy after portal vein embolization (PVE) based on clinical factors and radiomics of pretreatment computed tomography (CT)

**Patients and methods:**

Clinical information and CT scans of 241 consecutive patients from three Swedish centers were retrospectively collected. One center (120 patients) was applied for model development, and the other two (59 and 62 patients) as test cohorts. Logistic regression analysis was adopted for clinical model development. A FLR radiomics signature was constructed from the CT images using the support vector machine. A model combining clinical factors and FLR radiomics signature was developed. Area under the curve (AUC) was adopted for predictive performance evaluation

**Results:**

Three independent clinical factors were identified for model construction: pretreatment standardized FLR (odds ratio (OR): 1.12, 95% confidence interval (CI): 1.04–1.20), alanine transaminase (ALT) level (OR: 0.98, 95% CI: 0.97–0.99), and PVE material (OR: 0.27, 95% CI: 0.08–0.87). This clinical model showed an AUC of 0.75, 0.71, and 0.68 in the three cohorts, respectively. A total of 833 radiomics features were extracted, and after feature dimension reduction, 16 features were selected for FLR radiomics signature construction. When adding it to the clinical model, the AUC of the combined model increased to 0.80, 0.76, and 0.72, respectively. However, the increase was not significant.

**Conclusions:**

Pretreatment CT radiomics showed added value to the clinical model for predicting FLR hypertrophy following PVE. Although not reaching statistically significant, the evolving radiomics holds a potential to supplement traditional predictors of FLR hypertrophy.

**Supplementary Information:**

The online version contains supplementary material available at 10.1245/s10434-024-16592-z.

The main cause of mortality after major liver resection is posthepatectomy liver failure (PHLF) due to insufficient future liver remnant (FLR), i.e., the remaining part of the liver after surgery.^1,2^ To ensure safe surgery, portal vein embolization (PVE) has been established as a standard preoperative intervention to induce FLR hypertrophy in patients at risk, before major liver resection.^3^ By selectively blocking portal venous blood flow to the liver segments to be removed, a compensatory hypertrophy in the nonembolized segments is induced after PVE. PVE improves resectability of liver tumors by reducing the risk of PHLF. However, the response to PVE varies among patients and about one-third cannot proceed to liver resection due to insufficient FLR hypertrophy or tumor progression during the waiting period.^4^ Prediction of FLR growth response prior to PVE remains a significant challenge.

Many clinical factors have been identified to be associated with FLR hypertrophy following PVE, such as age, baseline liver function, the presence of underlying liver disease (for example, cirrhosis or steatosis), and nutritional status (e.g., sarcopenia).^5–7^ However, few studies have developed a prediction model based on these conventional clinical variables. In addition, although some novel methods have been proposed, they involve complex measures such as four-dimensional flow magnetic resonance imaging (MRI)^8^ or invasive procedures,^9^ limiting a wider application in clinical practice.

In recent years, radiomics has emerged as a powerful tool in medical image analysis.^10^ By converting routinely used medical images such as computed tomography (CT) into high-dimensional, mineable data, radiomics enables the capture of nuance information that may not be visible to the human eye.^11^ When coupled with advanced machine learning algorithms, these large numbers of quantitative features can be adopted to build prediction models for a variety of oncological applications, including tumor characterization, prognostication, and response to treatment prediction.^12,13^ With radiomics, the potential for individualized medical predictions may be significantly expanded.

This study hypothesized that radiomics can sensitively identify texture patterns associated with liver growth capacity from pretreatment CT images, which may be supplemental to clinical predictive factors for early readout of insufficient FLR hypertrophy after PVE. Therefore, this study aimed to propose a prediction model by combining clinical predictors and radiomics signature derived from CT scans for liver hypertrophy estimation in patients with liver malignancy subjected to PVE. Such a model can potentially aid surgeons in patient risk stratification and operative strategy selection.

## Patients and Methods

### Study Design and Patient Inclusion

**Fig. 1:**
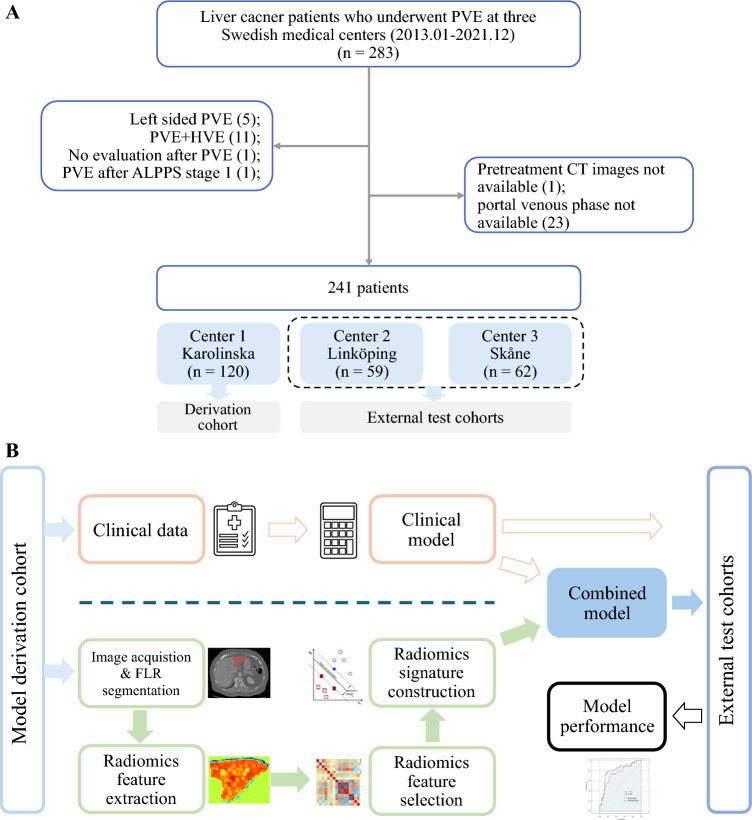
Patient selection process (**A**) and the prediction model building workflow (**B**). ALPPS, associating liver partition and portal vein ligation for staged hepatectomy; PVE, portal vein embolization; HVE, hepatic vein embolization

This multicenter, retrospective study included consecutive patients with liver malignancy intended for curative hepatectomy who received preoperative PVE. Patients were included from three Swedish medical centers, i.e., Karolinska University Hospital (center 1), Linköping University Hospital (center 2), and Skåne University Hospital Lund (center 3), between January 2013 and December 2021. Patients were excluded if they had: (1) left-sided PVE, (2) no imaging evaluation after PVE, (3) PVE plus hepatic vein embolization, (4) PVE after associated liver partition and portal vein ligation for staged hepatectomy (ALPPS) stage 1, and (5) no available pretreatment CT scans were obtained at portal venous phase. The patient selection process and the study workflow are shown in Fig. 1.

At each participating center, the decision to conduct PVE was determined by a multidiscipline tumor team, which comprehensively assessed pretreatment standardized FLR (pre-sFLR), the patient’s medical history and treatment history. For patients who had undergone chemotherapy, the pre-sFLR threshold was adjusted to 30%. In cases where patients had cirrhosis, the pre-sFLR threshold was established at 40%.^14^ The details of the PVE procedure have been described in previous work.^15^

According to our statistical power analysis, at least 100 patients were required to develop a prediction model achieving an AUC > 0.7 with a significance level (*α*) of 0.05 and a statistical power (1–*β*) of 0.90 when assuming an incidence of insufficient FLR hypertrophy of 30%. The insufficient FLR hypertrophy rate (30%) was based on literature and observational data.^4,16^ The significant difference in AUC between the models was set at 0.1. In this study, center 1 was used for model development (i.e., “derivation cohort”) and centers 2 and 3 for model performance evaluation (i.e., “external test cohorts”). During the process of model development, test cohorts were kept blind.

This study was approved by the Swedish Ethical Review Authority with approval number Dnr: 2019-01297. Informed consent was waived due to the retrospective nature of the study design. Patient information and image data were deidentified and analyzed in an anonymized manner.

### Clinical Factors and Liver Volume Calculation

Patient clinical data at baseline (prior to PVE) were retrieved from the hospital electronic records, including demographic information, body mass index (BMI), overall physical performance, chronic diseases, diagnosis, history of chemotherapy, laboratory tests (usually obtained the day before PVE), liver function evaluation, and PVE procedure-related information, such as embolization method and materials. For clinical variables with a missing rate ≤ 5%, the missing value was imputed by median for continuous variables and mode for categorical variables; variables with missing rate > 5% were abandoned.

Liver volume was calculated based on radiological imaging such as CT or MRI before and after PVE, according to local practice. The sFLR was determined by dividing the radiologically measured FLR with the estimated total liver volume, following the formula proposed by Vauthey:^17^ estimated total liver volume = − 794.41 + 1267.28 × body surface area. The body surface area was calculated using Mosteller’s formula.^18^ Kinetic growth rate (KGR) was determined as (post-sFLR-pre-sFLR)/time elapsed in weeks.

In this study, insufficient FLR hypertrophy was defined as post-sFLR < 30% after PVE at first imaging evaluation, considering that a majority of patients had undergone previous chemotherapy, and in the case of concomitant cirrhosis, a post-sFLR cutoff of 40% was applied.

### Clinical Model Development

Univariable logistic regression analysis was performed on clinical factors to detect the associations between them and FLR hypertrophy. Clinical factors with *p* < 0.1 were subjected to multivariable logistic regression analysis. The Akaike information criterion was employed to determine the number of predictors kept in the model. The clinical model was constructed by linear combination of these variables weighted by the corresponding coefficients.

### Radiomics Signature Development

#### CT Scan Acquisition and FLR Segmentation

Portal venous phase images of iodinated contrast-enhanced CT before PVE treatment were selected for radiomics analysis in this study. The scanning and reconstruction parameters included: tube voltage ranging from 70 to 120 kVp, tube current between 120 and 300 mAs, a pitch varying from 0.6 to 1.25 mm, an image matrix of 512 × 512, and a reconstruction slice thickness of 0.6–5 mm.

**Fig. 2:**
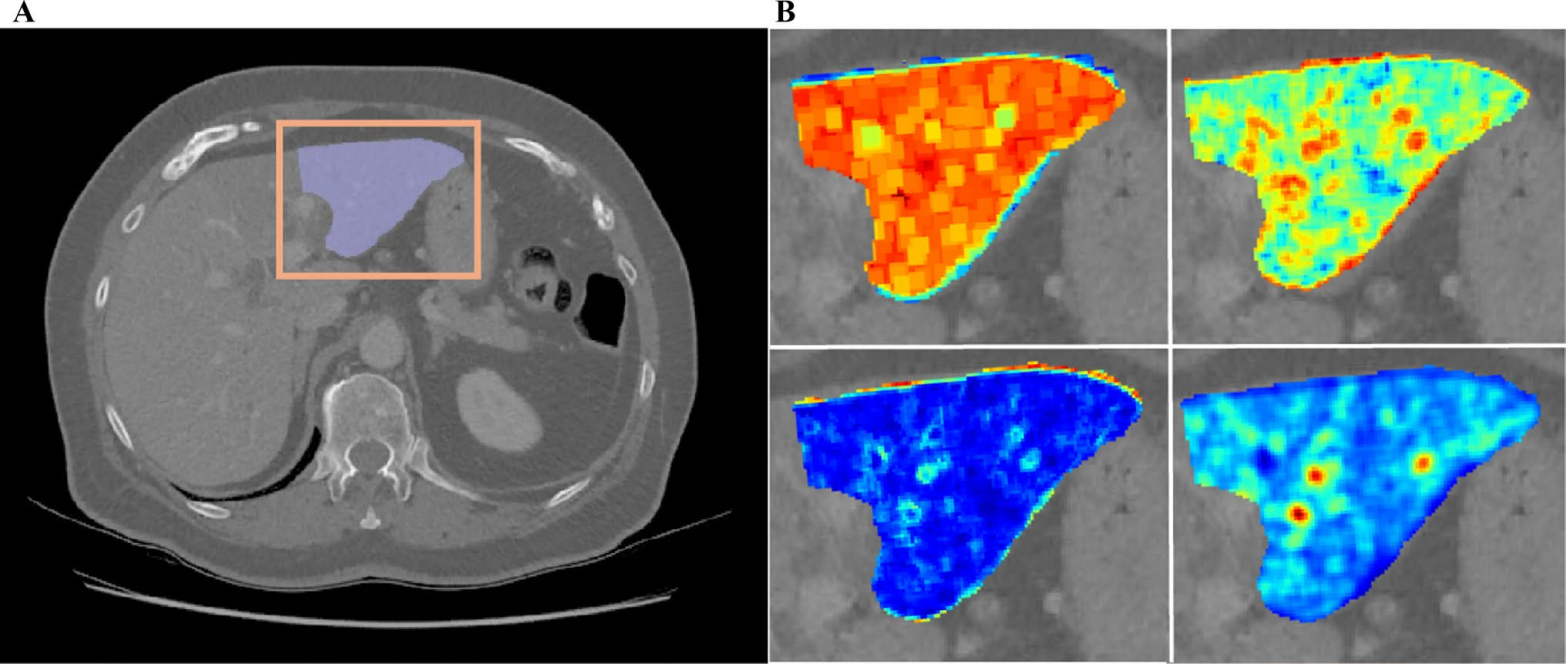
A representative case of CT study. **A** Radiomics features were extracted from the segmented future liver remnant part at the slice of the bifurcation level of the main portal vein. **B** Four representative radiomics features that show a texture pattern different from that perceived by human naked eye

As the CT images stemmed from different centers with various manufacturers and scanning protocols, imaging preprocessing was performed to standardize the images. The image voxel size was re-sampled to 1 × 1 × 1 mm^3^ by the B-spline approach and the intensity histogram was discretized using a fixed bin width of 25. After that, the FLR was manually segmented by one researcher (Q.W. with 6 years of abdominal imaging) at the portal vein bifurcation level of the cross-section CT image, by using ITK-SNAP (version 3.6.0) (Fig. 2). To evaluate reproducibility of radiomics features, 20 patients were randomly selected from the derivation cohort and delineated by another independent researcher. The interclass correlation coefficient (ICC) was calculated.

#### Radiomics Feature Extraction

Radiomics features were extracted from the segmented FLR by using the Python-based library pyradiomics (version 3.1.0). The following categories of radiomics features were extracted: shape, first order, gray level co-occurrence matrix, gray level dependence matrix, gray level run length matrix, gray level size zone matrix, and neighborhood gray tone difference matrix (Supplementary Table S1). The definitions of the radiomics features are available at https://pyradiomics.readthedocs.io/en/latest/features.html.

#### Radiomics Feature Selection

As the extracted radiomics features were high dimensional, feature selection was conducted to reduce potential overfitting when developing the model. Specifically, a “three-step” strategy was adopted: (1) radiomics features with ICC > 0.80 were selected, (2) Spearman correlation analysis between any two radiomics features was performed to compare the similarity of each feature pair (if the coefficient of the feature pair was larger than 0.95, one of them was randomly removed), and (3) Spearman correlation analysis was further conducted to evaluate the association between radiomics features and liver growth rate (i.e., KGR), and the significant radiomics features (*p* < 0.05) were chosen. With this “three-step” strategy, reproducible, non-redundant, and clinically significant radiomics features were chosen.

#### Radiomic Signature Construction

The radiomics features were normalized by *Z*-score before developing the radiomics signature. The Kruskal–Wallis test was performed, and the features were sorted by their

*F*-values. Support vector machine (SVM) was applied to develop the radiomics signature. The linear kernel function was adopted as it has good interpretability of the final model. To achieve a balanced classification between positive and negative samples, the synthetic minority oversampling technique (SMOTE) was adopted.^19^ To determine the hyperparameters of the model, a fivefold cross-validation was applied in the derivation cohort. The predicted probability was used as the FLR radiomics signature.

### Construction of Combined Model

The developed radiomics signature was incorporated into the clinical model to build a combined clinical–radiomics model. The performance of the combined model was evaluated and compared with the clinical model in both derivation and test cohorts.

### Statistical Analysis

Continuous variables are expressed as mean and standard deviation when the data was normally distributed and compared by Student’s *t*-test; when nonnormally distributed, they were expressed as median with interquartile range and compared by the Mann–Whitney *U* test. Categorical variables were presented as number and percentage and compared using chi-squired test or Fisher’s exact test.

The discriminative ability of the models was evaluated by the area under the curve (AUC), accuracy, sensitivity, specificity, and positive and negative predictive values. The calibration ability of the model was intuitively visualized by a calibration curve. The optimal threshold was determined by Youden’s index, which maximizes the sum of sensitivity and specificity. This optimal threshold of the combined model was also applied for patient risk stratification (low/high-risk group). A two-tailed *p* value less than 0.05 was regarded statistically significant. Statistical analysis and data visualization were conducted by using R software (version 4.2.1; R Foundation for Statistical Computing, Vienna, Austria) with corresponding packages. Sample size was estimated using PASS software (version 15.0.5; Utah, USA).

## Results

During the study period, 241 patients who underwent PVE were included in the study (120 in derivation cohort, and 59 and 62 in external test cohort 1 and 2, respectively). The main diagnosis was colorectal liver metastasis (114/241). The mean pre-sFLR was 24% (± 8%), which increased to 34% (± 11%) after a mean waiting time of 4.1 weeks. A total of 76 (32%) patients did not reach sufficient FLR hypertrophy after PVE (38, 19, and 19 patients in the three cohorts, respectively). There was no significant difference in insufficient FLR growth ratio among the three cohorts (*p* > 0.05). Detailed information on baseline characteristics in the separate cohorts is provided in Table 1.

**Table 1:** Basic characteristics of study subjects

	Derivation cohort (*n* = 120)	External test cohort 1 (*n* = 59)	External test cohort 2 (*n* = 62)	*p*-value
Sex				0.299
Male	80 (66.7%)	33 (55.9%)	42 (67.7%)	
Female	40 (33.3%)	26 (44.1%)	20 (32.3%)	
Age (years)	65.8 (10.8)	65.4 (10.1)	65.1 (10.9)	0.921
BMI	25.8 (4.2)	25.1 (4.2)	25.7 (3.7)	0.521
ECOG				< 0.001
0	41 (34.2%)	31 (52.5%)	11 (17.7%)	
1	72 (60.0%)	20 (33.9%)	46 (74.2%)	
2	7 (5.8%)	8 (13.6%)	5 (8.1%)	
ASA				0.042
1	21 (17.5%)	14 (23.7%)	9 (14.5%)	
2	65 (54.2%)	33 (55.9%)	25 (40.3%)	
3	34 (28.3%)	12 (20.3%)	28 (45.2%)	
Diabetes mellitus				0.211
No	104 (86.7%)	53 (89.8%)	49 (79.0%)	
Yes	16 (13.3%)	6 (10.2%)	13 (21.0%)	
Liver cirrhosis				0.005
No	119 (99.2%)	54 (91.5%)	62 (100%)	
Yes	1 (0.8%)	5 (8.5%)	0 (0.0%)	
Diagnosis				< 0.001
CRLM	47 (39.2%)	25 (42.4%)	42 (67.7%)	
BTC	68 (56.7%)	26 (44.1%)	14 (22.6%)	
HCC	2 (1.7%)	6 (10.2%)	6 (9.7%)	
Other^#^	3 (2.5%)	2 (3.4%)	0 (0%)	
Lesion number	1 [1–6]	1 [1–6]	2.5 [1–7]	0.051
Lesion diameter (mm)	29.5 [20.0–50.0]	66.0 [47.5–80.0]	34.0 [20.0–61.0]	< 0.001
No. of chemotherapy cycles	0 [0–5]	0 [0–2]	5 [0–6]	< 0.001
Bilirubin (mg/dL)	0.14 [0.07;0.27]	0.12 [0.08;0.28]	0.08 [0.06;0.11]	< 0.001
Albumin(g/dL)	3.09 (0.55)	3.28 (0.53)	3.60 (0.51)	< 0.001
AST (U/L)	37.7 [27.4–59.3]	44.3 [35.6–68.9]	33.8 [25.1–44.8]	0.019
ALT (U/L)	39.5 [26.3–78.0]	46.7 [29.3–71.9]	32.9 [20.7–49.7]	0.023
Gamma-GT (U/L)	120 [55.1–362]	162 [83.8–338]	86.8 [44.5–172]	0.011
Creatinine (umol/L)	76.2 (19.6)	79.3 (23.4)	74.0 (16.4)	0.342
Platelets (× 10^9^/L)	248 [210–310]	282 [212–382]	242 [191–319]	0.339
APTT(s)	25 [22–33]	26 [25–27]	26 [24–27]	0.880
INR	1.03 (0.11)	1.05 (0.11)	1.02 (0.11)	0.286
ALBI grade				< 0.001
Grade 1	7 (5.8%)	5 (8.5%)	21 (33.9%)	
Grade 2	76 (63.3%)	39 (66.1%)	38 (61.3%)	
Grade 3	37 (30.8%)	15 (25.4%)	3 (4.8%)	
MELD score				0.039
< 9	98 (81.7%)	44 (74.6%)	57 (91.9%)	
≥ 9	22 (18.3%)	15 (25.4%)	5 (8.1%)	
Pre-sFLR(%)	21.5 (6.7)	28.4 (10.9)	23.7 (8.1)	< 0.001
PVE material				< 0.001
Microparticles	88 (73.3%)	3 (5.1%)	0 (0.0%)	
NBCA	32 (26.7%)	56 (94.9%)	62 (100%)	
PVE method				0.918
Contralateral	6 (5.0%)	2 (3.4%)	2 (3.2%)	
Ipsilateral	114 (95.0%)	57 (96.6%)	60 (96.8%)	
rPVE+S4				0.024
No	86 (71.7%)	50 (84.7%)	54 (87.1%)	
Yes	34 (28.3%)	9 (15.3%)	8 (12.9%)	
Degree of hypertrophy (%)	9.1 [4.5–12.7]	12.2 [7.2–16.5]	9.0 [5.5–13.4]	0.034
Kinetic growth rate (%/w)	2.5 [1.4–3.8]	3.6 [1.8–6.1]	2.6 [1.7–4.0]	0.018
Insufficient FLR growth				0.982
No	82 (68.3%)	40 (67.8%)	43 (69.4%)	
Yes	38 (31.7%)	19 (32.2%)	19 (30.6%)	

^*^Statistical significance

^#^Include gastrointestinal stroma cell tumor, anal cancer metastasis, neuroendocrine tumor, malignant melanoma metastasis

*APTT* activated partial thromboplastin time, *ASA* American Society of Anesthesiologists Classification, *AST* aspartate transaminase, *ALBI* albumin–bilirubin grading system, *ALT* alanine transaminase, *BMI* body mass index, *BTC* biliary tract cancer, *CRLM* colorectal liver metastasis, *ECOG* Eastern Cooperative Oncology Group, *HCC* hepatocellular carcinoma, *INR* international normalized ratio, *MELD* model for end-stage liver disease, *PVE* portal vein embolization, *rPVE* right-sided PVE, *pre-sFLR* standardized future liver remnant before PVE treatment, *NBCA*, *n*-butyl cyanoacrylate

### Clinical Model

Univariable logistic regression analysis identified four clinical factors that significantly correlated with insufficient FLR hypertrophy: pre-sFLR, ALT level, embolization material, and method. According to the minimum Akaike information criterion in multivariable logistic regression, three independent clinical factors remained for modelling, i.e., pre-sFLR (OR: 1.12; 95% CI: 1.04–1.20), ALT level (OR: 0.40; 95% CI: 0.18–0.86), and PVE material (OR: 0.25; 95% CI: 0.04–1.65) (Table 2). When incorporating these three factors as a clinical model, it yielded an AUC of 0.75 in derivation cohort, 0.71 in test cohort 1, and 0.68 in test cohort 2 (Table 3).

**Table 2:** Logistic regression analysis of clinical variables with insufficient liver hypertrophy after portal vein embolization in derivation cohort

	Univariable logistic regression				Multivariable	
	Sufficient growth (*n* = 82)	Insufficient growth (*n* = 38)	OR	*p*-value	OR	*p*-value
Sex				0.33		
Male	57 (69.5%)	23 (60.5%)	Ref.			
Female	25 (30.5%)	15 (39.5%)	1.49 (0.67−3.32)			
Age (years)	65.5 (10.9)	66.3 (10.6)	1.01 (0.97–1.04)	0.71		
BMI	25.5 (3.78)	26.3 (4.89)	1.04 (0.95–1.14)	0.36		
ECOG						
0	29 (35.4%)	12 (31.6%)	Ref.			
1	49 (59.8%)	23 (60.5%)	1.13 (0.49–2.62)	0.77		
2	4 (4.88%)	3 (7.89%)	1.81 (0.35–9.35)	0.48		
ASA						
1	14 (17.1%)	7 (18.4%)	Ref.			
2	47 (57.3%)	18 (47.4%)	0.77 (0.27–2.21)	0.62		
3	21 (25.6%)	13 (34.2%)	1.24 (0.4–3.87)	0.71		
Diabetes mellitus				0.54		
No	70 (85.4%)	34 (89.5%)	Ref.			
Yes	12 (14.6%)	4 (10.5%)	0.69 (0.21–2.29)			
Liver cirrhosis				0.99		
No	82 (100%)	37 (97.4%)	Ref.			
Yes	0 (0.00%)	1 (2.63%)	Inf (0–Inf)			
Diagnosis						
CRLM	30 (36.6%)	17 (44.7%)	Ref.			
BTC	49 (59.8%)	19 (50.0%)	0.68 (0.31–1.52)	0.35		
HCC	0 (0.00%)	2 (5.26%)	Inf (0–Inf)	0.99		
Other^#^	3 (3.66%)	0 (0.00%)	0 (0–Inf)	0.99		
Lesion number	3.94 (4.98)	4.47 (5.50)	1.02 (0.95–1.1)	0.59		
Lesion diameter	34.8 (24.1)	36.5 (22.2)	1 (0.99–1.02)	0.73		
No. of chemotherapy cycles	1.82 (2.71)	1.81 (3.19)	1 (0.86–1.16)	0.99		
Bilirubin (mg/dL)	32.1 (54.1)	17.7 (17.5)	0.37 (0.1–1.38)	0.14		
Albumin (g/dL)	30.7 (5.42)	31.3 (5.68)	1.24 (0.61–2.53)	0.55		
AST (U/L)	0.94 (0.69)	0.75 (0.63)	0.99 (0.98–1)	0.17		
ALT (U/L)	1.22 (1.32)	0.68 (0.48)	0.98 (0.97–0.99)	0.02*	0.98 (0.97–0.99)	0.02*
Gamma-GT (U/L)	4.88 (6.03)	4.29 (6.92)	1(1–1)	0.63		
Creatinine (umol/L)	77.4 (19.2)	73.5 (20.2)	0.99(0.97–1.01)	0.31		
Platelets (×10^9^/L)	280 (119)	252 (71.6)	1 (0.99–1)	0.18		
APTT(s)	39.1 (101)	28.8 (6.79)	1 (0.99–1.01)	0.63		
INR	1.03 (0.11)	1.02 (0.12)	0.71 (0.02–21.84)	0.84		
ALBI grade						
Grade 1	4 (4.88%)	3 (7.89%)	Ref.			
Grade 2	50 (61.0%)	26 (68.4%)	0.69 (0.14–3.33)	0.65		
Grade 3	28 (34.1%)	9 (23.7%)	0.43 (0.08–2.29)	0.32		
MELD score				0.43		
<9	66 (80.5%)	32 (84.2%)	Ref.			
≥9	16 (19.5%)	6 (15.8%)	0.77 (0.28–2.16)			
pre-sFLR (%)	20.4 (5.79)	23.9 (7.80)	1.08 (1.02–1.15)	0.01*	1.12 (1.04–1.20)	< 0.01*
PVE material				0.07*		0.03*
Microparticles	56 (68.3%)	32 (84.2%)	Ref.			
NBCA	26 (31.7%)	6 (15.8%)	0.40 (0.15–1.08)		0.27 (0.08–0.87)	
PVE method				0.08*		0.15
Contralateral	2 (2.44%)	4 (10.5%)	Ref.			
Ipsilateral	80 (97.6%)	34 (89.5%)	0.21 (0.04–1.22)		0.25 (0.04–1.65)	
rPVE+S4				0.23		
No	56 (68.3%)	30 (78.9%)	Ref.			
Yes	26 (31.7%)	8 (21.1%)	0.57 (0.23–1.42)			

^*^Statistical significance

^#^Include gastrointestinal stroma cell tumor, anal cancer metastasis, neuroendocrine tumor, malignant melanoma metastasis

*ASA* American Society of Anesthesiologists Classification, *BMI* body mass index, *BTC* biliary tract cancer, *CRLM* colorectal liver metastasis, *ECOG* Eastern Cooperative Oncology Group, *HCC* hepatocellular carcinoma, *MELD* model for end-stage liver disease, *PVE* portal vein embolization, *pre-sFLR* standardized future liver remnant before *PVE* treatment, *ALBI* albumin-bilirubin grading system, *rPVE* right sided PVE, *NBCA n*-butyl cyanoacrylate, *OR* odds ratio.

**Table 3:** Performance metrics of the developed models in three cohorts

	Model	AUC (95% CI)	ACC	Sens	Spec	PPV	NPV
Clinical model	Derivation cohort	0.75 (0.66–0.85)	0.74	0.74	0.74	0.57	0.86
	Test cohort 1	0.71 (0.54–0.88)	0.83	0.64	0.95	0.88	0.81
	Test cohort 2	0.68 (0.53–0.84)	0.73	0.61	0.79	0.64	0.77
Combined model	Derivation cohort	0.80 (0.71–0.89)	0.8	0.79	0.80	0.65	0.89
	Test cohort 1	0.76 (0.61–0.90)	0.78	0.65	0.85	0.68	0.82
	Test cohort 2	0.72 (0.57–0.87)	0.76	0.64	0.82	0.67	0.80

*ACC* accuracy, *AUC* area under the curve, *Sens* sensitivity, *Spec* specificity, *PPV* positive predictive value, *NPV* negative predictive value

### Radiomics Signature Construction

A total of 833 radiomics features were extracted from the CT portal venous phase images. The distribution of feature category is described in Supplementary Fig. S1. After removing the features with ICC < 0.80, 701 reproducible radiomics features were retained. In the Spearman correlation analysis, 380 highly correlated features (*r* > 0.95) were filtered out. After that, the 94 radiomics features with significant correlation with KGR were selected for modeling. Finally, 16 radiomics features were determined by the Kruskal–Wallis test and fed into the SVM classifier. Radiomics signature was constructed accordingly. The information on the SVM hyperparameters and the 16 features is provided in supplementary Table S2.

### Combined Model and Its Performance

The developed combined model, incorporating the radiomics signature into the clinical model, is expressed as

$$\begin{aligned} Y\, = \, & 1.10 \times {\text{pre}} - {\text{sFLR}}\,\left( \% \right) + 0.33 \times {\text{PVE}}\,{\text{material}}\,\left( {{\text{microparticle}}:\,0,\,{\text{NBCA}}:\,1} \right) \\ & + 0.99 \times {\text{ALT}}\,{\text{level}}\,\left( {\text{U/L}} \right) + 13.3 \times {\text{FLR}}\,{\text{radiomics}}\,{\text{signature}} \\ \end{aligned}$$

**Fig. 3:**
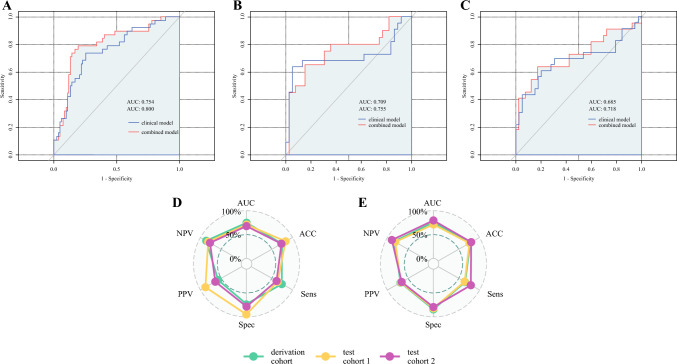
Predictive performance of the developed models. Area under the curve of the clinical model and the clinical–radiomics model in the derivation (**A**) and test cohorts (**B**, **C**); predictive performance metrics of the clinical (**D**) and the combined clinical–radiomics model (**E**). ACC, accuracy, AUC, area under the curve; Sens, sensitivity; Spec, specificity; PPV, positive predictive value; NPV, negative predictive value

**Fig. 4:**
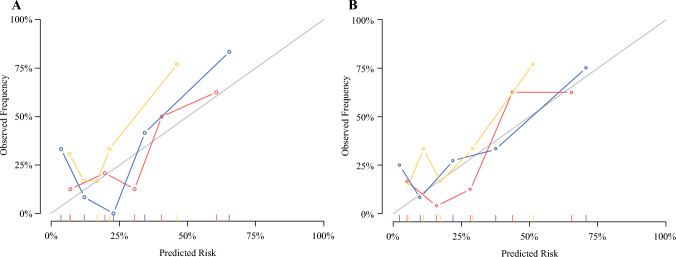
Calibration curves of the developed models. The clinical model (**A**) and the clinical–radiomics model (**B**) in the derivation (red line) and test cohorts (blue and yellow lines for test cohort 1 and 2, respectively). The diagonal line represents an ideal situation where the model predicted probability fits perfectly the actual observed risk, indicating that, the closer to the diagonal line, the better a prediction model is

The combined clinical–radiomics model improved the predictive performance for predicting insufficient FLR hypertrophy. Compared with the clinical model, the clinical–radiomics model showed better fitting (Supplementary Table S3). The AUC increased from 0.75 to 0.80 in the derivation cohort, from 0.71 to 0.76 in test cohort 1, and from 0.68 to 0.72 in test cohort 2 (Fig. 3). Although not statistically significant according to the Delong test (*p* > 0.05), the obtained AUC was slightly higher. The optimal threshold of the combined model was determined as 0.372. Detailed information on other measures can be found in Table 3. Calibration curves showed consistency between the observed and expected outcome rates in both derivation and test cohorts (*p* > 0.05) (Fig. 4).

**Fig. 5:**
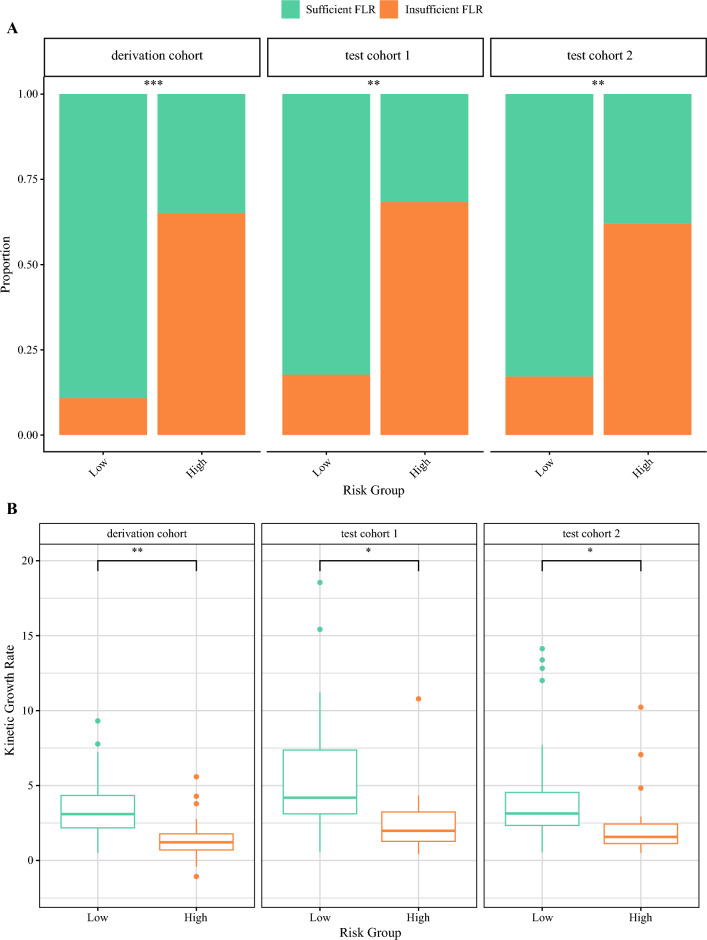
Comparison of the risk groups stratified by the combined model. Clinical outcome (sufficient or insufficient FLR hypertrophy) in different risk groups across the three cohorts (**A**). The difference in kinetic growth rate in different risk groups was significant across three cohorts (**B**). **p < 0.01; ***p < 0.001

To facilitate the clinical utility, patients were stratified into different risk groups (low or high) by the optimal threshold of the combined model (i.e., 0.372). The proportion of insufficient FLR hypertrophy and the KGR were significantly different between the two risk groups among the three cohorts (*p* < 0.05) (Fig. 5).

## Discussion

This study developed a combined model based on three baseline clinical variables (pre-sFLR, embolization material, and ALT level) and CT-derived radiomics signature for predicting insufficient FLR hypertrophy following PVE in patients with liver tumors. The model yielded an AUC of 0.80 in the derivation cohort and showed robust predictive power in two external test cohorts with an AUC of 0.76 and 0.72. Although the combined model showed improved AUC values over the clinical model, the difference did not achieve statistical significance.

The identification of pre-sFLR as a predictive variable is consistent with previous studies,^20–22^ highlighting its importance in predicting post-PVE liver hypertrophy. In a systematic review that comprehensively evaluated clinical risk factors of insufficient FLR hypertrophy, 13 out of 18 included studies (72%) stated the indicative value of pre-sFLR^7^ A lower pre-sFLR is associated with better postprocedure hypertrophy of the nonembolized liver segments with a pooled correlation coefficient of − 0.37.^7^

The choice of embolization material plays a crucial role in PVE efficacy, as different materials can influence the extent of embolization and subsequent liver hypertrophy. Several studies have investigated and compared the difference between embolization materials, specifically *n*-butyl-cyanoacrylate glue (NBCA) and microparticles. NBCA has shown overall superiority over microparticles in terms of FLR hypertrophy, procedural safety, and radiation exposure. Our findings confirmed the observed differences of the materials in introducing FLR hypertrophy, with three times higher risk of insufficient growth after microparticles compared to glue. Notably, our model showed robust performance in external cohorts where the predominant embolization materials were used differently from the derivation cohort.

Serum ALT level, which is a common indicator of liver injury or inflammation, was detected as an independent factor for FLR hypertrophy following PVE. Such a finding has not been reported before. In the clinical context, it is reasonable as liver injury/inflammation potentially undermines liver regeneration. In a single-center study with 152 patients, Watanabe et al. detected two serum enzymes (alkaline phosphatase and choline esterase) as significant indicators of insufficient FLR hypertrophy after PVE.^20^ The independent role of these enzymes requires further verification. Interestingly, the commonly used clinical scoring systems for liver function evaluation, such as albumin–bilirubin grading system (ALBI) or model for end-stage liver disease (MELD) score, did not show a significant correlation with FLR hypertrophy. As it was significant on both uni- and multivariable logistic regression analyses, ALT was incorporated into the combined model as a continuous variable.^23^ The combined model provides insights into factors potentially influencing liver regeneration following PVE. Further studies are needed to capture additional clinical and biological dimensions.

An extensive body of studies have demonstrated the utility of radiomics in various oncological and nononcological settings, suggesting that radiomics can uncover clinically relevant information that complements conventional clinical assessments.^24–26^ Several studies have shown CT texture information indicative of liver function. Studies by Wu et al. and Zhu et al. showed that pretreatment CT radiomics-based models can accurately predict indocyanine green retention level in hepatocellular carcinoma patients, aiding in the noninvasive evaluation of hepatic functional reserve.^27,28^ Another study, which combined CT radiomic features from nontumoral liver parenchyma (referred to as  virtual biopsy) with clinical data, significantly improved preoperative risk assessments for liver resections, e.g., to predict postoperative liver dysfunction.^29^

To date, very few studies have applied radiomics in the PVE setting. In a single-center, retrospective study, Gerwing and colleagues extracted radiomics features from the liver, spleen, and bone marrow from portal venous phase CT images for model construction.^30^ Their model showed an AUC of 0.88 for predicting sufficient FLR hypertrophy probability. In another study, a similar approach was applied to construct an artificial neural network model based on texture features of the CT portal venous phase images, which reached an AUC of 0.75 in the prediction of FLR hypertrophy response.^16^

While promising, these studies are limited by small sample size (53 and 55 patients, respectively) and lack of external validation, which implies potential overfitting in their models. Our study extends the evidence by using an expanded cohort size and two independent validation cohorts, demonstrating the applicability of CT radiomics in predicting insufficient FLR hypertrophy after PVE. Our study included a larger number of radiomics features and a more extensive feature selection process, which contributed to a robust analysis of FLR hypertrophy predictors. Our study also provided further insight into how a combined model integrating clinical and imaging factors can be applied to support clinical decision-making. While the combined model showed higher predictive measures than the clinical model, this improvement did not reach statistical significance. Notably, radiomics remains an evolving field, with ongoing studies being devoted to refine feature selection, standardize protocols, and improve the interpretability of radiomics-based models. It holds significant promise in oncological and nononcological fields.

The CT radiomics signature developed in this study contributed additional information to the clinical factor-derived model for predicting FLR hypertrophy after PVE. While the combined model showed improved fitting metrics compared with the clinical model alone, the increase in predictive accuracy, as measured by AUC, was not statistically significant. This finding suggests that, while radiomics may add delicate details, further studies with larger sample sizes are necessary to confirm any significant improvement in predictive performance.

The utilization of CT images is particularly pertinent in this study, as CT is a standard part of the preoperative assessment in liver surgery. It is also a commonly used examination when evaluating volumetric change after PVE. In other words, developing a radiomics signature does not increase the examination burden on patients. By leveraging advanced imaging analysis, clinicians can obtain a more nuanced understanding of liver-specific factors influencing liver regeneration. This approach aligns with the broader trend of incorporating AI and machine learning into clinical decision-making processes.

There are some limitations in this study. First, the retrospective nature may introduce selection bias, and prospective validation is required to confirm our findings. Second, there were several clinical variables that were not “balanced” among the three cohorts, which were considered to be consistent with the real-world situation. For example, owing to center preference, microparticles were seldomly used in the two test cohorts, while they were preferably used in the derivation cohort (*p* < 0.05) during the study period. As this study aimed to develop a model targeting routinely clinical application, statistical techniques were not adopted to balance these variables. Third, although external validation was performed in two independent Western cohorts with fair performance, the study population may still not fully represent the diverse clinical scenarios encountered in routine practice or in other ethnicities. Further validation in larger, multicenter cohorts, such as the international DRAGON cohort,^31,32^ is necessary. Last, other reported clinical risk factors were not evaluated in the derivation cohort, such as body composition parameters, which may also contribute to poor FLR hypertrophy. Besides, owing to the limited sample size and patient and treatment predisposition, several established indicators were not detected as significant in our cohort and therefore not included in our models.

Future research should focus on addressing these limitations and further refining the predictive model. Prospective studies with larger sample sizes and diverse patient populations and scenarios (such as liver deprivation technique) are essential to validate the clinical utility of our clinical–radiomic model. Investigating the integration of other imaging modalities, such as MRI, with radiomics could provide additional predictive value. Moreover, exploring the underlying biological mechanisms driving the radiomics features could enhance the interpretability of the models.

In conclusion, when incorporating radiomics signature into a clinical model, it enhances the predictive accuracy for insufficient FLR volume growth following PVE, although it did not reach a significant level. Radiomics may offer supplementary insights into traditional predictors of predicting FLR hypertrophy. Our findings align with the growing body of literature supporting the integration of radiomics and clinical data for predictive modeling.

## Supplementary Information

Below is the link to the electronic supplementary material.

Supplementary file1 (DOCX 78 kb)

## Data Availability

The original contributions presented in the study are included in the article/Supplementary Materials. Further inquiries can be directed to the corresponding author.
