# Supplementary material for: Development and External Validation of a Combined Clinical–Radiomic Model for Predicting Insufficient Hypertrophy of the Future Liver Remnant following Portal Vein Embolization
Source: Ann Surg Oncol. 2024 Dec 10;32(3):1795–807. doi: 10.1245/s10434-024-16592-z (PMC11811440; doi:10.1245/s10434-024-16592-z)

Development and external validation of a combined clinical-radiomic model for predicting insufficient hypertrophy of the future liver remnant following portal vein embolization

**Supplementary materials**

**Table S1.** Radiomics feature category

| Feature category | Number |
| --- | --- |
| Original | 105 (12.6%) |
| Wavelet | 728 (87.4%) |
| Shape | 14 (1.7%) |
| First order | 162 (19.4%) |
| Gray-level co-occurrence matrix (glcm) | 198 (23.8%) |
| Gray-level dependence matrix (gldm) | 126 (15.1%) |
| Gray-level run length matrix (glrlm) | 144 (17.3%) |
| Gray-level size zone matrix (glszm) | 144 (17.3%) |
| Neigbouring Gray Tone Difference Matrix (ngtdm) | 45 (5.4%) |

Note: Detailed definitions of each category can be found at https://pyradiomics.readthedocs.io/en/latest/features.html

**Table S2.** Hyper-parameters of the support vector machine classifier for FLR radiomics signature construction:

kernel=’ linear’, degree = 3, C = 1.0, probability =True; others used as default.

| Radiomics features | Coefficient |
| --- | --- |
| original_firstorder_Kurtosis | -0.283 |
| original_glcm_ClusterProminence | -0.224 |
| original_glcm_Idmn | 1.064 |
| original_glcm_InverseVariance | 0.486 |
| original_gldm_GrayLevelNonUniformity | 0.412 |
| original_glszm_SizeZoneNonUniformityNormalized | -0.488 |
| original_shape_Maximum2DDiameterSlice | 0.123 |
| wavelet-HHH_glszm_ZoneEntropy | -0.433 |
| wavelet-HHL_firstorder_10Percentile | -0.068 |
| wavelet-HHL_firstorder_RootMeanSquared | 0.586 |
| wavelet-HLL_firstorder_10Percentile | 0.303 |
| wavelet-HLL_glcm_InverseVariance | -0.600 |
| wavelet-LHH_glszm_GrayLevelNonUniformityNormalized | -0.117 |
| wavelet-LHH_glszm_ZoneEntropy | -0.127 |
| wavelet-LHL_glcm_Imc1 | 0.741 |
| wavelet-LLH_glszm_SizeZoneNonUniformityNormalized | 0.634 |

**Table S3**. Model fitting comparison

| Model | AIC | BIC | R2 | adjusted R2 | RMSE |
| --- | --- | --- | --- | --- | --- |
| Clinical model | 147.730 | 161.667 | 0.147 | 0.125 | 0.430 |
| Combined model | 142.957 | 159.682 | 0.194 | 0.166 | 0.418 |

*Note: AIC, Akaike Information Criterion; BIC, Bayesian Information Criterion; R² coefficient of determination; RMSE, root mean squared error. Generally, for AIC, BIC and RMSE, the smaller indicates the model better, while R2 and adjusted R2 the opposite.*

Figure S1: Distribution of radiomics features extracted from CT image


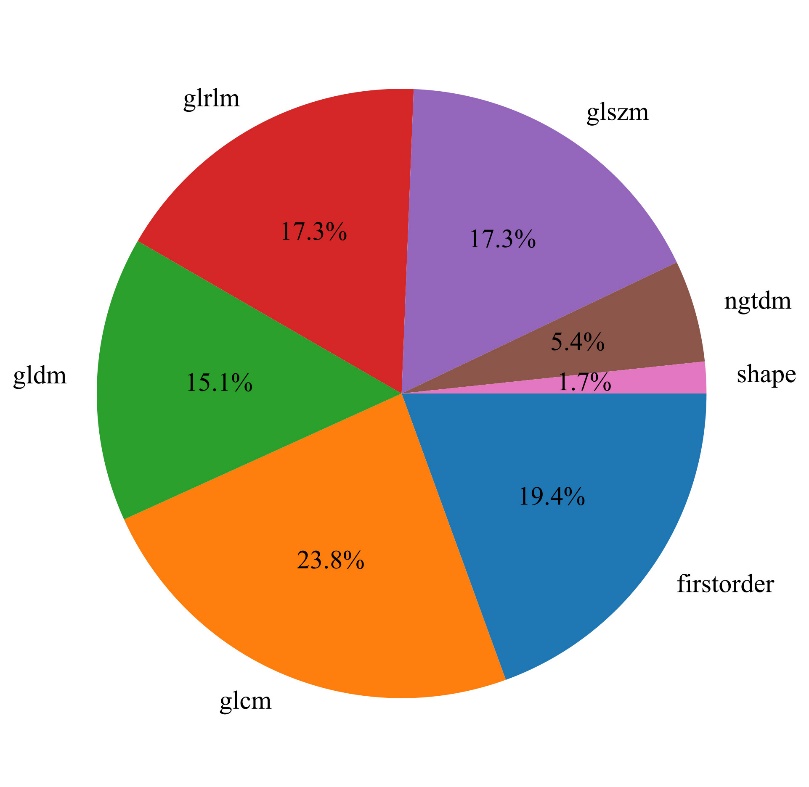

Supplement: Supplementary file 1 — Supplementary file1 (DOCX 78 kb) [file 10434_2024_16592_MOESM1_ESM.docx]
